# Synthesis and *in vitro* study of surface-modified and anti-EGFR DNA aptamer -conjugated chitosan nanoparticles as a potential targeted drug delivery system

**DOI:** 10.1016/j.heliyon.2024.e38904

**Published:** 2024-10-05

**Authors:** Maryam Rahmani Kheyrollahi, Javad Mohammadnejad, Akram Eidi, Hanieh Jafary

**Affiliations:** a Department of Biology, Science and Research Branch, Islamic Azad University, Tehran, P.O. Box 14515/775, Iran; b Department of Life science engineering, Faculty of New Sciences and Technologies, University of Tehran, 1439957131, Tehran, Iran

**Keywords:** Chitosan nanoparticles, DNA-Aptamer, Controlled noscapine release, Targeted drug delivery, Cancer cell inhibition

## Abstract

Nowadays, finding effective approaches for cancer therapy is one of the significant issues related to human health all over the world. Hence, in this research, we designed and synthesized a novel targeted DDS based on surface-modified chitosan (CS) for the effective delivery of noscapine (NO). As the surface of CS nanoparticles was firstly modified with carboxyl groups and followed by covalent conjugation of DNA-aptamer (Ap) as targeting and receptor blocker agent. Secondly, NO, as a chemotherapeutic agent, was loaded into prepared nano-complex and synthetics were effectively characterized via various analytical devices, including FT-IR, 1H NMR, DLS, Zeta potential analyzer, TGA, TEM, and SEM to verify quality and quantity of synthetics. Drug loading was obtained about 25 % and sustained drug release was observed for nano-complex at different pHs. Then, the cell viability assay was performed on MCF-7 (as breast cancer cell) and HFF-1 (as normal cell) cell lines to investigate cancer cell inhibition potency of nano-complex. Cell viability of cancer cells was 19.84 ± 1.87 % (for C-CS-Ap-NO) and 75.43 ± 2.64 % (for C-CS-Ap) after 72 h of treatment with 400 nM concentration. These results have been confirming the excellent potency of synthesized novel nano-complex as practical DDS in cancer therapy.

## 1 Introduction

Cancer is one of the most common causes of death all over the world and its mortality has been increasing at a galloping rate despite major advances in the understanding of tumor biology [1,2]. As 1806590 cancer cases have been predicted in 2020 in the United States according to the American Cancer Society report [3]. Among various types of cancers, breast cancer (as a leading cause of cancer death among females) has high morbidity and incidence among women throughout the world; about 276480 new cases of invasive and 48530 new cases of non-invasive breast cancer are estimated to be diagnosed among women in the United States in 2020 [1,4]. Therefore, cancer therapy is a prominent issue related to human health all over the world that should solve. On the other hand, conventional therapeutic approaches such as chemotherapy, radiation therapy, surgery, and so forth led to the fast development of cellular resistance and as well as toxic side effects on the normal cells of the whole body owing to lack of specificity [5,6]. Additionally, the risks of therapeutic failure and thereby tumor relapse have considerably enhanced due to using less efficient conventional approaches in cancer therapy [7]. As a result, the need for declining the harmful side effects of the above-mentioned methods and rising the efficacy of most antitumor therapeutic agents by improving their accumulation inside cancer cells have been remarkably increased [8]. In order to eliminate these hurdles, appropriate cancer therapy approaches such as novel drug delivery systems (DDSs) are needed to improve cancer therapy efficiency. Among these novel methods, nanoparticle-based DDSs can intensively apply for cancer diagnosis and therapy since they have several benefits such as sustained drug release manner in the tumor site, improve drug stability in the systemic circulation, eliminate adverse side effects targeted accumulation in cancer cells, and so forth which made them as a promising candidate for cancer cell growth inhibition [[9], [10], [11]].

Among various nanoparticle-based carriers which have been used for DDSs goals, chitosan (CS; poly [β-(1–4)-linked-2-amino-2-deoxy-D-glucose]) nanoparticles because of their various advantages, including non-immunogenicity, biocompatibility, biodegradability, and so forth are known as an excellent candidate for efficient drug delivery to cancer cells [12,13]. The practical biomedical applications of CS nanoparticles are also in reconstructive medicine, tissue engineering, wound healing, etc. [14]. Other aspects of CS nanoparticles are excellent bioavailability, good solubility, great serum stability and hydrophilicity, suitable pharmacodynamics and pharmacokinetic that make them effectively appropriate for cancer therapy [15]. The surface amino groups of these nanoparticles led to their excellent capacity in surface modification and surface engineering which, in turn, provides an opportunity for their surface decorations. Conjugation of various ligands such as antibody, folic acid, peptide, aptamer, and so on is the practical part of nanoparticle-based pharmaceutics by which smart, biocompatible, and stable novel DDSs can be fabricated [16]. By decoration of the surface of CS nanoparticles, they can remarkably develop to use in targeted DDSs in which ligands can act as targeting agents of cancer cells’ receptors that are called receptor-mediated or active internalization [17].

Among different targeted ligands, nucleic acid aptamers (Ap: kind of oligonucleotide) have been interestingly using for targeted DDSs in both cancer diagnosis and therapy. The unique, stable, and three-dimensional single-stranded DNA or RNA Aps which fold into certain structures can target their receptors on the surface of cancer cells with high binding affinity and specificity [18,19]. For instance, a specific kind of aptamer can effectively attach to epidermal growth factor receptor (EGFR), blocks it, and as well as act as EGFR targeting. EGFR is overexpressed in a wide variety of solid tumors such as epidermoid carcinomas, lung and liver cancer, malignant gliomas, and breast cancers [20,21]. Besides, since using anti-EGFR antibodies such as cetuximab and panitumumab led to primary or acquired resistance in patients EGFR-targeting by anti-EGFR aptamer can significantly improve the efficiency of EGFR blocking [22]. Hence, anti-EGFR aptamer not only can act as a targeting agent in DDSs, but it also can act as a drug and blocks its receptor on the surface of cancer cells. On the other hand, therapeutic agents such as noscapine (NO) can load into nanocarrier for further cancer cell growth inhibition. This therapeutic drug can act as efficient antiangiogenic and anticancer agent and subsequently leads to cell cycle arrest and induces apoptosis in cancer cells [23,24]. NO blocks microtubule in cancer cells and interferes in their cell divisions which, in turn, activates the mitotic checkpoints to stop the cell cycle. Furthermore, NO down-regulates hypoxia-mediated HIF-1α expression, induces PTEN/PI3K/mTOR signaling pathway, reduces the secretion of the potent angiogenic VEGF cytokine, and consequently, leads to cell death in cancer tissues [25].

In the present study, we firstly developed a novel carboxyl-modified and single-strand DNA-Ap-functionalized CS nano-complex which was followed by loading of noscapine in it. The synthesized samples, then, effectively characterized by analytical devices, including FT-IR, ^1^H NMR, DLS, TGA, and TEM. Excellent drug loading capacity and *in vitro* drug release profile were obtained for nano-complex. Afterward, cancer cell inhibition potency of CS derivatives was implemented.

## 2 Materials and methods

### 2.1 Chemicals and apparatus

CS (Mw: 50000–190000 Da; Viscosity: 20–300 cp), sodium tripolyphosphate (TPP), 1-Ethyl-3-(3-dimethyl-aminopropyl) carbodiimide (EDC), N-hydroxysuccinimide (NHS), di-methyl-sulphoxide (DMSO), and cellulose dialysis membranes were purchased from Sigma Aldrich. Also, 3-(4,5-dimethylthiazol-2-yl)-2,5-diphenyltetrazolium bromide (MTT), the RPMI 1640 medium, and fetal bovine serum (FBS) were obtained from Sigma-Aldrich. The high-performance liquid chromatography (HPLC)-purified anti-EGFR DNA aptamer (5′-TGA ATG TTG TTT TTT CTC TTT TCT ATA GTA-3′) was synthesized by Sangon Biotech Co., Ltd. (Shanghai, China). The MCF-7 breast cancer cell line was bought from the Institute of Pasteur, Iran.

Spectroscopic assessments of syntheses were performed using a NanoDrop 2000c UV–Vis spectrophotometer (Thermo scientific), Fourier transform infrared (FT-IR) spectrometer (PerkinElmer 843), and proton nuclear magnetic resonance (^1^H NMR) (Bruker Avance III 400 MHz, Germany). Transmission electron microscopy (TEM) images were taken by means of Philips EM 208S (Netherlands) and dynamic light scattering (DLS) tests were carried out by NanoBrook 90 Plus (Brookhaven, USA). Moreover, the TGA analyses were conducted by STA 1500, Rheometric Scientific.

### 2.2 Synthesis of carboxylated-chitosan

To synthesis carboxylated-chitosan (C-CS), the CS solution was prepared as the following procedure: 200 mg of CS was solubilized in 6 mL of acetic acid and was stirred overnight at room temperature. After that, the NaOH (1 mM) solution was added to the CS-containing solution drop by drop to obtain CS precipitation. Then, CS precipitation was filtered and washed with deionized water three times. Afterward, CS solution was submerged in 20 mL of isopropanol for further washing which was followed by adding 50 mg of NaOH salt in it under vigorous stirring at room temperature for 5 h. In the next section, C-CS was synthesized as the following approach; 8 mg of chloroacetic acid was suspended in 5 mL isopropanol and was slowly added to the previously prepared CS solution for 15 min under stirring at 50 rpm. The prepared solution was stirred for 10 h at the light-protected condition and room temperature. Then, the C-CS was washed with a mixture of water and ethanol (3:1 ratio) three times and saved at 4 °C.

### 2.3 Synthesis of aptamer-conjugated chitosan

In this phase, DNA aptamer was conjugated to C-CS by esterification reaction with NHS in the presence of 1-EDC which was followed by amid bond formation between the aptamer and modified CS. As 5 mg of aptamer were practically dissolved in 10 ml of deionized water. On the other hand, 40 ml of 0.4 mM EDC solution and 40 ml of 0.1 mM NHS solution were prepared and mixed. This solution act as an activator of C-CS. Afterward, the NHS/EDC mixture was added to the prepared C-CS solution (100 mg C-CS solubilized in 15 mL of deionized water) under stirring at 60 rpm for 4 h. Thereafter, the prepared aptamer solution was added to the activated C-CS mixture drop by drop and was stirred at 60 rpm for 24 h at room temperature. These reactions were performed under nitrogen atmosphere and dark circumstances. Eventually, the Ap-C-CS nano-complex was purified by dialysis tube in deionized water (4 times) for 24 h to separate excessive and unreacted reactants. The prepared nano-complex was recovered for analytical characterization and biomedical tests by lyophilizing and saving at 4 °C.

### 2.4 Drug loading into Ap-C-CS nano-complex

The noscapine (NO) as a kind of effective chemotherapeutic agent was loaded in the Ap-C-CS nano-complex by the equilibrium dialysis method. Briefly, 5 mg (6 × 10^−8^ M) of the NO was dissolved in 5 mL of deionized water under stirring at 80 rpm for 5 h to obtain a homogenous solution. Thereafter, the drug-containing solution was added to 5 mL of Ap-C-CS (3 × 10^−4^ M) at 37 °C and was stirred overnight at 90 rpm with a stirrer. Then, 1 mL of TPP was slowly added to the drug-containing solution under ultra-sonication conditions to form globular nano-complex. Subsequently, the drug-loaded nano-complex was spilled in the dialysis membrane and followed by placing in 1000 ml of the PBS buffer (pH 7.4) at 37 °C on a magnetic stirrer for 10 min to separate unloaded chemotherapeutic agents. The purification was repeated with deionized water at the same condition.

### 2.5 Characterization of syntheses

#### 2.5.1 FT-IR analysis

In order to qualify the surface modification of CS, conjugation of the DNA aptamer to the C-CS by the formation of amide bonds, and verifying successful drug loading into nano-complex the FT-IR was performed in the transmittance mode, using the pellet procedure with KBr, in the wavelength range of 400–4000 cm^−1^.

#### 2.5.2 ^1^H NMR analysis

^1^H NMR spectroscopy of CS derivatives was implemented applying D_2_O (as the solvent) and methanol (as the co-solvent) with 400 MHz at room temperature and the location of peaks for various samples and conjugations was drawn using MestReNova software.

#### 2.5.3 TGA analysis

The TGA technique was carried out for the measurement of thermal stability of CS conjugations and drug loading capacity of Ap-C-CS nano-complex. This analysis was done under a nitrogen atmosphere. As 5 mg of specimens was heated from 30 °C to 600 °C at a heating rate of 10 °C/min in a pan under a constant nitrogen flow rate (40 mL/min).

#### 2.5.4 DLS analysis

DLS technique was applied to determine the average hydrodynamic size of the specimens. As the freshly prepared samples were vigorously sonicated using an ultrasonication device at 37 °C for 15 min to equilibrate the temperature. Then, the size measurements were performed at 37 °C in a quartz cuvette at nM concentrations.

#### 2.5.5 TEM analysis

The particle size and morphology of the CS derivatives were observed by TEM. As freshly prepared suspensions of the Ap-C-CS and drug-loaded nano-complexes were dispersed onto a copper grid and followed by its drying at 25 °C. Then, the excess liquid was absorbed using a filter paper, and subsequently, the sample images were taken at an accelerating voltage of 120 kV.

#### 2.5.6 SEM analysis

The surface morphology and distribution of CS synthetics were investigated through SEM. As samples were firstly fixed on the stub against carbon adhesive tape. Then, the specimens were sputter-coated with gold and subjected to SEM at 25 kV for analysis.

### 2.6 Drug release study

The *in vitro* drug release patterns of Ap-C-CS-NO and CS-NO nano-complexes were assessed in phosphate buffer saline (PBS) as the release medium with different pHs (7.4, 6.0, and 5.0) at 37 °C. As 4 mg of synthesized Ap-C-CS-NO nano-complex was firstly solubilized in 4 ml of PBS and spilled in the prepared dialysis tube. Secondly, the dialysis bag was submerged in 50 ml of the release medium (PBS) at a backer and the whole system was placed in an incubator-shaker apparatus at 50 rpm at 37 °C. Thereafter, 150 μL of the release medium was collected at predetermined intervals of time up to 64 h, and the removed release medium was replaced with fresh medium at all times. Subsequently, drug content was analyzed based on a free drug calibration curve using UV–Vis spectrophotometry at a wavelength of 210 nm. The release experiments were repeated in triplicate. This scenario was separately carried out for three different pHs.

### 2.7 Cell culture

The human MCF-7 breast cancer cell line and human HFF1 foreskin fibroblast normal cells were bought from the Pasture Institute of Iran. These cell lines were separately cultured in the RPMI medium (Gibco, UK) supplemented with 10 % FBS, 2 mM glutamine, 100 μgmL^−1^ streptomycin, and 100 IU mL^−1^ penicillin for cellular analysis.

### 2.8 Cell viability assay

The cell growth inhibitory effects of CS, C-CS, Ap-C-CS, Ap-C-CS-NO, and pure NO with 100 nM, 200 nM, and 400 nM concentrations on MCF-7 and HFF1 cells were assessed applying the MTT assay at 24 h, 48 h, and 72 h as the following procedure [26,27]. Briefly, the samples were prepared in a serum-supplemented tissue culture medium and sterilized using 0.2 mm filtration at pH 7.4. Then, 5000 cells/100 μL of both cancerous and normal cells were seeded in each well of 96-well microtiter plates and allowed to attach overnight. Thereafter, the culture medium of the microtiter plates was replaced by100 μL of serial dilutions of various prepared-specimens and the cells were incubated for 24 h. Afterward, the medium of sample-treated wells was replaced by 200 μL RPMI without serum. Subsequently, 20 μL of sterile-filtered MTT (3-[4,5-dimethylthiazol-2-yl]-2,5-diphenyl tetrazolium bromide) salt was dispersed in PBS pH 7.4 (5 mg/ml) and added to each well at 0.5 mg MTT/ml final concentration and incubated for another 4 h. The suspension liquid was removed and the cells were re-suspended in DMSO and optical density (OD) was read at 570 nm. The experiments were repeated in triplicate. The difference in OD values between the treated and non-treated cells was depicted as the value of cell viability.

### 2.9 Cellular uptake

In this section, the MCF-7 breast cancer cells were maintained at 37 °C (5 % CO_2_) in DMEM (Gibco, UK) medium supplemented with 10 % (v/v) FBS, 100 U/mL penicillin, and 1 mg/mL streptomycin. Thereafter, 100 nM of Annexin V-binding V-FITC-Ap-C-CS nano-complex was treated with 1 × 10^6^ cells/3 mL of MCF-7 cells in 6-well plates at 37 °C for 40 min. The cancerous cells were washed with PBS three times and the cells were gathered and resuspended in PBS buffer. Eventually, MCF-7 cells were subjected to FACS Calibur device (BD Biosciences, CA, USA) for evaluation of internalization amounts.

### 2.10 Statistical analyses

The experiments were repeated at least three times and results were reported as mean ± standard error of the mean. A comparison of each group was assessed by one-way analysis of variance (ANOVA) and T-test and differences were considered significant for ∗p < 0.05.

## 3 Results and discussion

### 3.1 FT-IR analyses

**Fig. 1:**
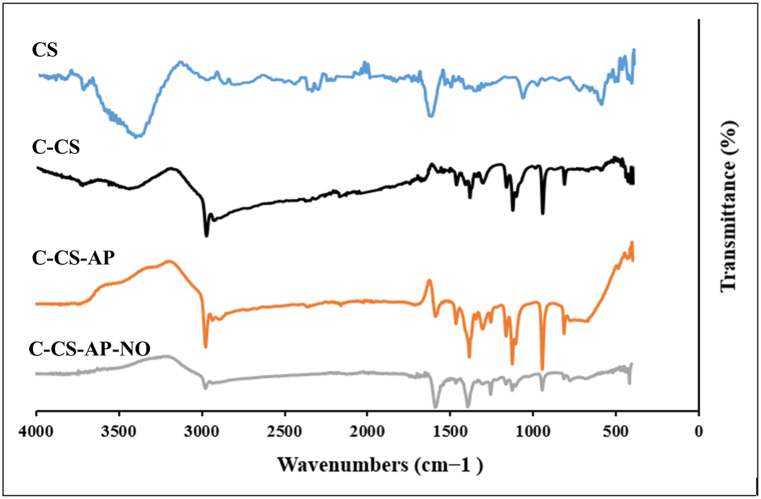
The FT-IR spectra of CS and its derivatives. The absorption peaks are presenting the appropriate surface modification, conjugation of Ap to C-CS, and presence of NO drug in nano-complex which confirms the excellent qualification of syntheses. For the CS sample, the peaks were detected at around 3437 cm^−1^ (–NH_2_ and –OH groups stretching vibration), 3337 cm^−1^ (O−H and N−H stretching vibrations), 1151 cm^−1^ (asymmetric stretching of −C−O−C− bridge), and 1034 cm^−1^ (C−N bond). Also, the sharp absorption peaks at around 1420 cm^−1^ to 1495 cm^−1^ could be due to the formation of amides bending (CH−NH−C=O) between amine groups of Ap and carboxyl groups of C-CS in the structure of Ap-C-CS nano-complex.

FT-IR spectroscopic analysis was implemented to verify the synthesis quality of CS nanoparticles and their derivatives (Fig. 1). The spectra of these samples were depicted as the following. For the CS sample, the peaks were detected at around 3437 cm^−1^ (–NH_2_ and –OH groups stretching vibration), 3337 cm^−1^ (O−H and N−H stretching vibrations), 1151 cm^−1^ (asymmetric stretching of −C−O−C− bridge), and 1034 cm^−1^ (C−N bond) [28]. The peaks at around 1490 cm^−1^ are attributed to the C–H stretching in methyl groups and other prominent peaks between 2800 cm^−1^ and 3000 cm^−1^ are owing to CH, CH_2_, and CH_3_ stretching vibrations in CS specimen. The absorption peaks at 1639 cm^−1^, 1388 cm^−1^, and 1068 cm^−1^ are assigned to C=O, C−N stretching, and C−O stretching vibrations in CS. On the other hand, the characteristic peaks at 2923 cm^−1^ to 2875 cm^−1^ are related to C-H stretching vibrations in C-CS. Also, the peak at around 1708 cm^−1^ verifies the presence of the carboxyl group in C-CS [29]. The sharp absorption peaks at around 1420 cm^−1^ to 1495 cm^−1^ could be due to the formation of amides bending (CH−NH−C=O) between amine groups of Ap and carboxyl groups of C-CS in the structure of Ap-C-CS nano-complex. For Ap-C-CS-NO nano-complex, the characteristic peaks at about 2949 cm^−1^, 2853 cm^−1^, 2701 cm^−1^ are indicative of O−CH_3_ and O−CH_2_ groups in the structure of NO. Furthermore, other absorption peaks of Ap-C-CS-NO are attained at around 1380 cm^−1^, and 1032 cm^−1^ which are due to the presence of N−CH_3_ and substituted benzene in the structure of NO, respectively [30].

### 3.2 ^1^H NMR analyses

**Fig. 2:**
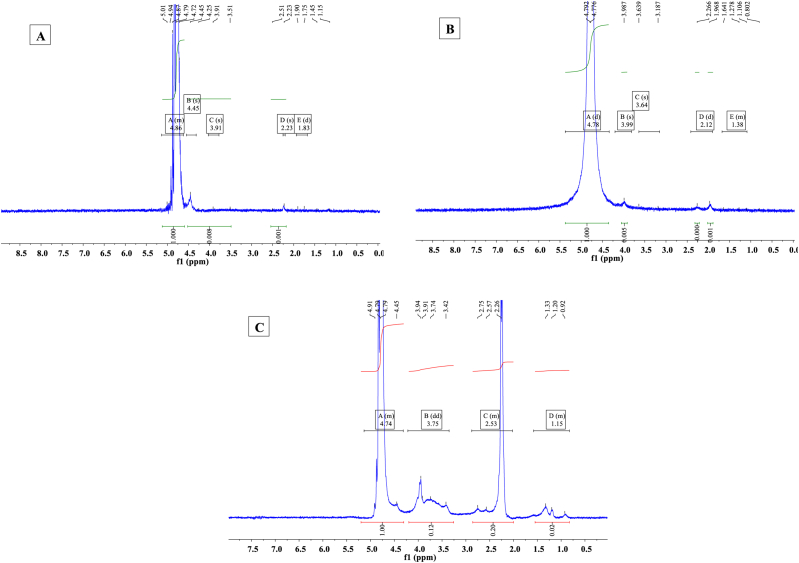
^1^H NMR spectra of CS and its derivatives. (A) ^1^H NMR of C-CS indicates resonance peaks from 3.00 ppm to 5.00 ppm which are assigned to the protons of glucosamine unit and the peaks at around 1.9 ppm and 2.23 ppm are corresponding to the methyl protons of the N-acetyl group in CS. Also, the peak around 1.2 ppm demonstrates the presence of CH_3_ of the ester group. (B) ^1^H NMR of C-CS-Ap shows the methyl proton peaks of Ap between 0.8 ppm and 1.96 ppm and as well as ethyl protons from 2.26 ppm to 2.75 ppm due to the presence of Ap in the structure of nano-complex. (C) ^1^H NMR of C-CS-Ap-NO nano-complex.

In the ^1^H NMR spectrum of C-CS, the appearance of peaks around 1.15 ppm and 1.45 ppm demonstrates the presence of CH_3_ of the ester group (Fig. 2). The resonance peaks from 3.51 ppm to 5.00 ppm are related to the protons of the glucosamine unit and the peaks at around 1.9 ppm and 2.23 ppm are corresponding to the methyl protons of the N-acetyl group in CS [31,32]. On the other hand, the methyl protons of Ap are detected between 0.8 ppm and 1.96 ppm and as well as ethyl proton are assigned from 2.26 ppm to 2.75 ppm due to the presence of Ap and NO in the structure of nano-complex which verifies the appropriate conjugation of Ap to C-CS and presence of the drug in nano-complex. Besides, the proton peaks in the spectrum of 0.8 ppm–2.06 ppm and as well as 3.18 ppm–3.63 ppm are assigned to aliphatic protons of amide bond formation (CH−NH−C=O) in the structure of Ap-C-CS-NO nano-complex [33]. These data have been demonstrating effective surface modification, surface functionalization, and the presence of NO in the compartment of synthetics.

### 3.3 DLS analyses

**Fig. 3:**
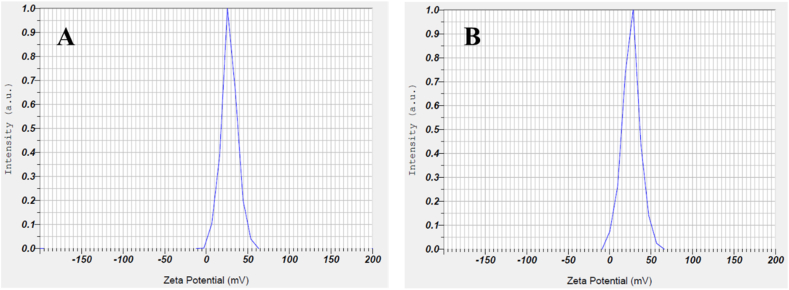
Zeta potential values of synthetic nano-complexes. The surface charge of C-CS-Ap (A) and C-CS-Ap-NO (B) formulations are at about +27.7 mV and +26.0 mV, respectively.

The hydrodynamic diameter and polydispersity index (PDI) of CS and its derivatives and as well as their surface changes were measured by means of the DLS. According to these results, the CS nanoparticle size was ascertained to be 56.5 nm (PDI: 0.13) in diameter. After surface modification of CS, the average size of the C-CS was indicated a slight increase and reached 76.5 (PDI: 0.19) with a good monodispersity. The main growth in hydrodynamic diameter of nano-complex was measured after conjugation of Ap (177 nm; PDI: 0.16) which indicating efficient covalent conjugation while no significant change was detected after drug loading into nano-complex that is indicating NO molecules were trapped inside globular C-CS-Ap nanocarrier. A minor increase in C-CS-Ap-NO size (189 nm; PDI: 0.17) may be due to the formation of non-covalent interaction between drug and the surface of nano-complex which, in turn, leads to a gradual increase of size. The particle size is one of the most important issues in the appropriate delivery of therapeutic agents to cancer cells since the physical stability, escaping from the immune system, using drug dosage, and effective therapeutic performance of the nano-complex is directly related to particle size [34]. Besides, PDI values for all samples were measured less than 0.2 that demonstrating great monodispersity without aggregation for synthetics. The monodispersity of nanocarrier is also crucial for its serum stability [35]. On the other hand, Zeta potential values of C-CS-Ap and C-CS-Ap-NO samples were determined at about +27.7 mV and +26.0 mV, respectively (Fig. 3). It is crystal clear that NO leads to the decline of surface charge of C-CS-Ap-NO nano-complex which is maybe due to the ionization of oxygen in carbonyl groups. High zeta potential values could result in good stability and repellence between the nano-complexes [36]. The positive surface charge of the samples could be owing to the positive amine groups of chitosan which are appropriate for effective internalization in cancer cells. As a matter of fact, the electrostatic interaction between the positive charge of nano-complex and negative charge of glycoproteins on the surface of cancer cells can lead to cellular uptake of nanocarrier [37].

### 3.4 TEM and SEM analyses

**Fig. 4:**
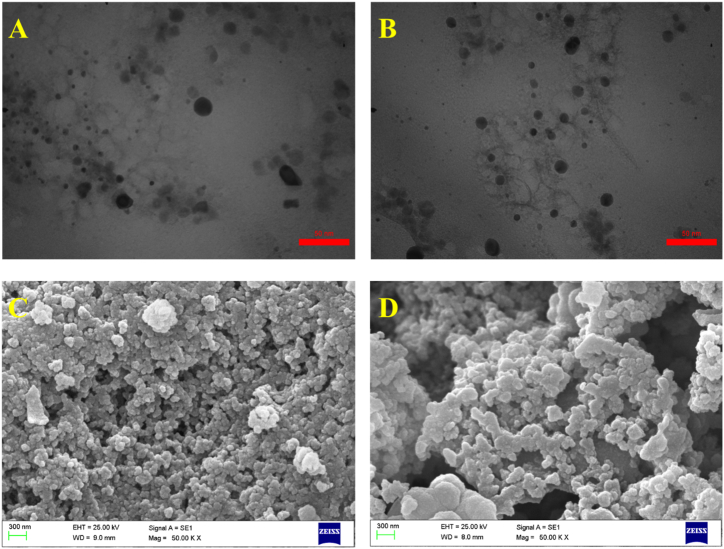
TEM images of CS derivatives, including C-CS-Ap (A) and C-CS-Ap-NO (B). These images are showing the nano-size, spherical, and globular structures with good dispersion for CS derivatives. The SEM images are also presented a suitable globular morphology and appropriate dispersion for C-CS-Ap (C) and C-CS-Ap-NO (D) nano-complexes.

The TEM and SEM were applied to take a visual image of C-CS-Ap and C-CS-Ap-NO nano-complexes for verifying the shape, structure, dispersion, and morphology of synthetics (Fig. 4). According to TEM images, both C-CS-Ap and C-CS-Ap-NO have a narrow particle size distribution with an average size of 30 nm–50 nm which are effectively appropriate for targeting cancer cells. Additionally, the gelatinous layers around the core of nano-complexes are due to conjugation of Ap to C-CS and they verify the presence of Ap in the nanocarrier compartment. Nanocarriers with a size less than 200 nm are reported to have good distribution inside capillaries of cancer tissues [3,38]. Moreover, nano-complexes sized 70–200 nm could have appropriate blood circulation and excellent cancer cell targeting via enhanced permeability and retention (EPR) effect [39]. On the other hand, the C-CS-Ap and C-CS-Ap-NO formulations indicate an almost spherical shape with a smooth surface in the SEM images. These images were also demonstrated to be of about 50 nm–80 nm in diameter which is in line with TEM results. Some irregular shapes may be due to the aggregation of nano-complexes since their lyophilized forms were used for SEM images. The nanometric size (less than 100 nm) of synthesized nano-complexes is favorable for DDS goals since the size cut-off of tumor vasculature is about 200 nm–700 nm [4,10].

### 3.5 TGA analyses and drug loading capacity

**Fig. 5:**
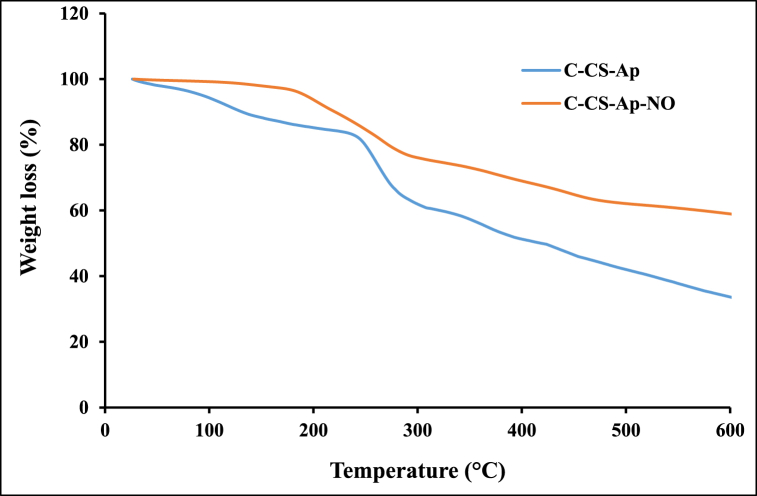
TGA analyses of the C-CS-Ap and C-CS-Ap-NO samples. As shown, the TGA curve of C-CS-Ap specimen has two main weight loss phases: 5 % at 110 °C and 28 % at around 250 °C–290 °C. The weight loss curve of the C-CS-Ap-NO sample was earned in the main phase between 185 °C and 287 °C. Based on these two curves, the drug content of nano-complex was calculated at about 25 %.

The physical state, thermal stability, and drug loading capacity of C-CS-Ap and C-CS-Ap-NO conjugates were investigated and measured through weight loss values using TGA (by the heating-cooling process) [40]. This analysis was done within the temperature range 30 °C–600 °C and the shift changes in the peaks of samples were used to measure loaded drug in C-CS-Ap nano-complex. According to the TGA curve of the C-CS-Ap specimen, the weight loss values were attained in two phases (Fig. 5). The first one was at about 5 % at 110 °C which was owing to water evaporation of nano-complex. The second weight loss was earned at around 250 °C–290 °C (about 28 %). On the other hand, the weight loss curve of the C-CS-Ap-NO sample was observed in the main phase between 185 °C and 287 °C (about 20 %). These data have been depicting appropriate thermal stability for both samples which is the main issue for the down-stream process of drug production at the industrial level. Additionally, by comparing the weight loss values of TGA curves of these two C-CS-Ap and C-CS-Ap-NO nano-complexes, the NO loading was calculated around 25 % which is considerable values for nanoparticle-based DDSs. High drug loading values play a significant role in effective drug delivery to cancer cells and as well as reduce the treatment periods of patients which is crucial for effective cancer therapy [8,41].

### 3.6 Drug release profile

**Fig. 6:**
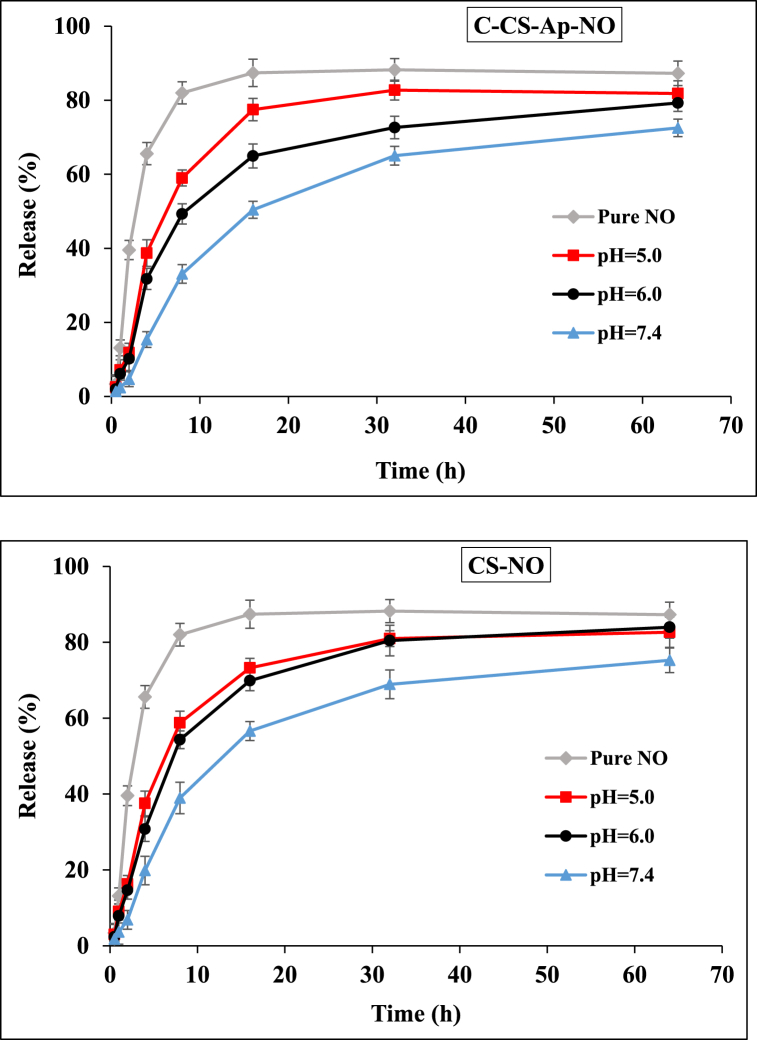
The drug release profile of pure NO, NO of C-CS-Ap-NO, and CS-NO nano-complexes under pH values of 7.4, 6.0, and 5.0. C-CS-Ap-NO sample shows a controlled release manner at pH 7.4 (as a mimic of physiologic medium) than pHs 6.0 and 5.0. The protonation of the CS chains at pH 6.0 and 5.0 is the main reason for the fast drug release rate that is appropriate for the effective delivery of NO to cancer cells since their environments and especially their endosomal organelles have the acidic condition. Besides, C-CS-Ap-NO indicates the controlled drug release manner than CS-NO specimen which could be due to its surface functionalization with Ap and as well as the surface modification.

The *in vitro* drug release profiles of pure NO, C-CS-Ap-NO, and CS-NO nano-complexes were performed at different pHs (7.4, 6.0, and 5.0) and outputs were shown the pH-sensitive drug release as indicated in Fig. 6. The PBS solution was applied to investigate the stability of the nano-complexes in a physiological condition. Furthermore, since the pH values of the endocytic compartments of cancer cells are ranged from 4.5 to 6.5 which is lower than the pH of normal physiological environment (pH 7.4), the drug release manner of nano-complexes was scrutinized at acidic pHs to appropriate comparison of cancer cells endosomes [8,42]. As indicated in Fig. 5, the burst drug release was obtained within 8 h at a peak of 33.1 % for C-CS-Ap-NO nano-complex at pH 7.4 while the release was 46.3 % and 59.01 % at pHs 6.0 and 5.0 at the same time, respectively. Moreover, more than 50 % of drug release was earned at 16 h whereas the release amounts were approximately 65 % and 77 % for C-CS-Ap-NO sample at the same time respectively, which is revealing the effect of acidic pHs in fast drug release performance of nano-complex. The final drug release value of C-CS-Ap-NO was also lower than both acidic pHs. By comparing these release values for nano-complex at various pHs with pure NO release profile, the controlled drug release manner of synthetic nano-complex is discernible. For example, while only 33.1 % of NO was released from nano-complex after 8 h under pH 7.4, the release value was 81.99 % (more than 2 folds of release in comparison to synthetic nano-complex) for pure NO under the same circumstances. Furthermore, the CS-NO sample was indicated a little bit faster drug release than C-CS-Ap-NO formulation at all times; for instance, 38.96 % of NO release was observed at the first 8 h and pH 7.4 which was higher than (about 5 %) of its release from C-CS-Ap-NO at the same conditions. The slow drug release of C-CS-Ap-NO versus CS-NO may be owing to the surface functionalization and modification of the C-CS-Ap-NO sample since surface carboxylation of CS and the presence of Ap sequences can act as cover for globular CS nanoparticle and prevent the fast rate of drug release. For the controlled drug release manner of nano-conjugate, various intermolecular and intramolecular interactions such as the electrostatic interactions, hydrogen-bonding, and π-π interactions can play the main role which leads to slow drug release from nano-complex. On the other hand, the acidic pH could result in protonation of the CS chain which, in turn, affects the drug interactions in the nano-complex formulation and results in fast drug release [43]. Additionally, the controlled release behavior of the C-CS-Ap-NO at physiological pH is stating that synthesized formulation can be stable in physiologic serum of the human body which is a crucial issue for effective drug delivery to target tissues and cancer cells [42].

### 3.7 Cell viability assay

**Fig. 7:**
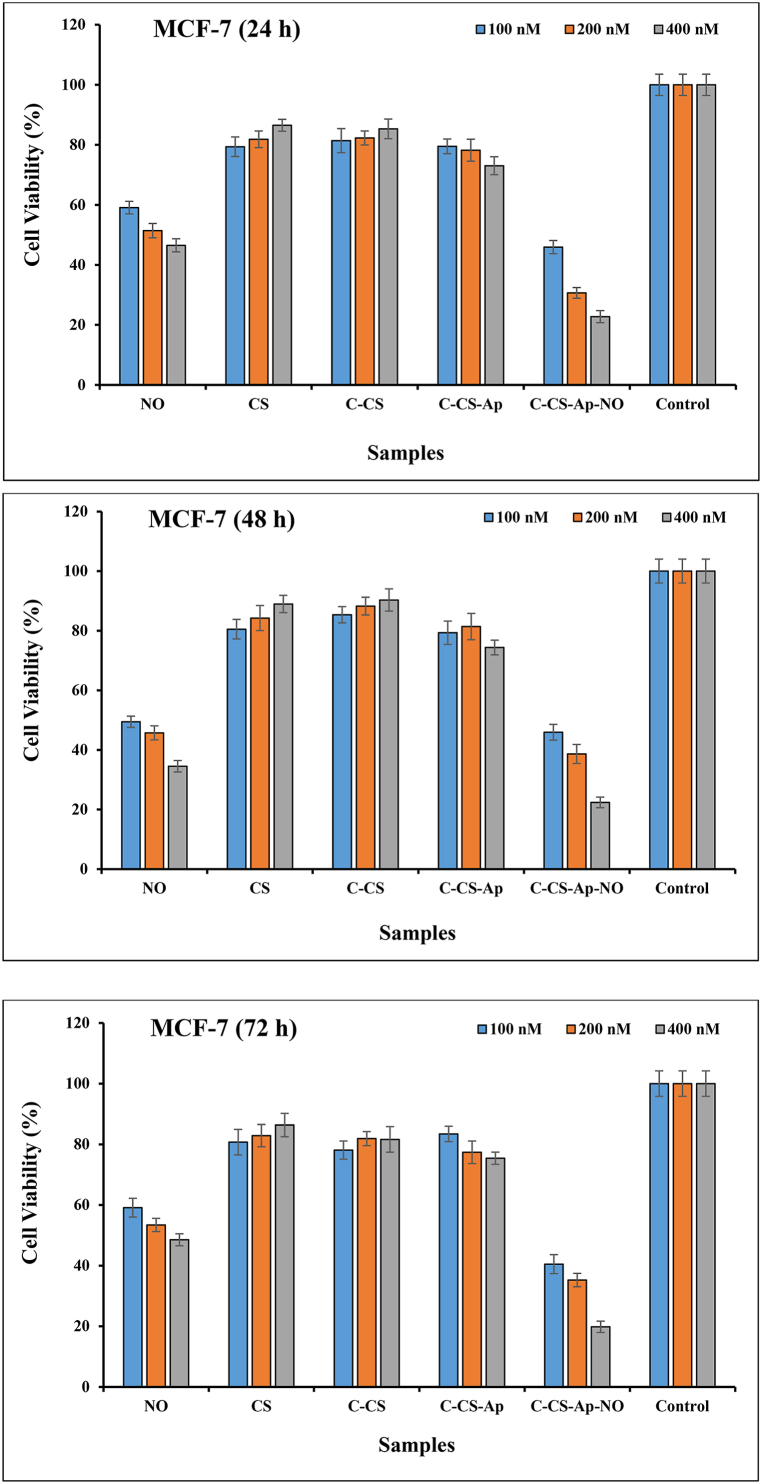

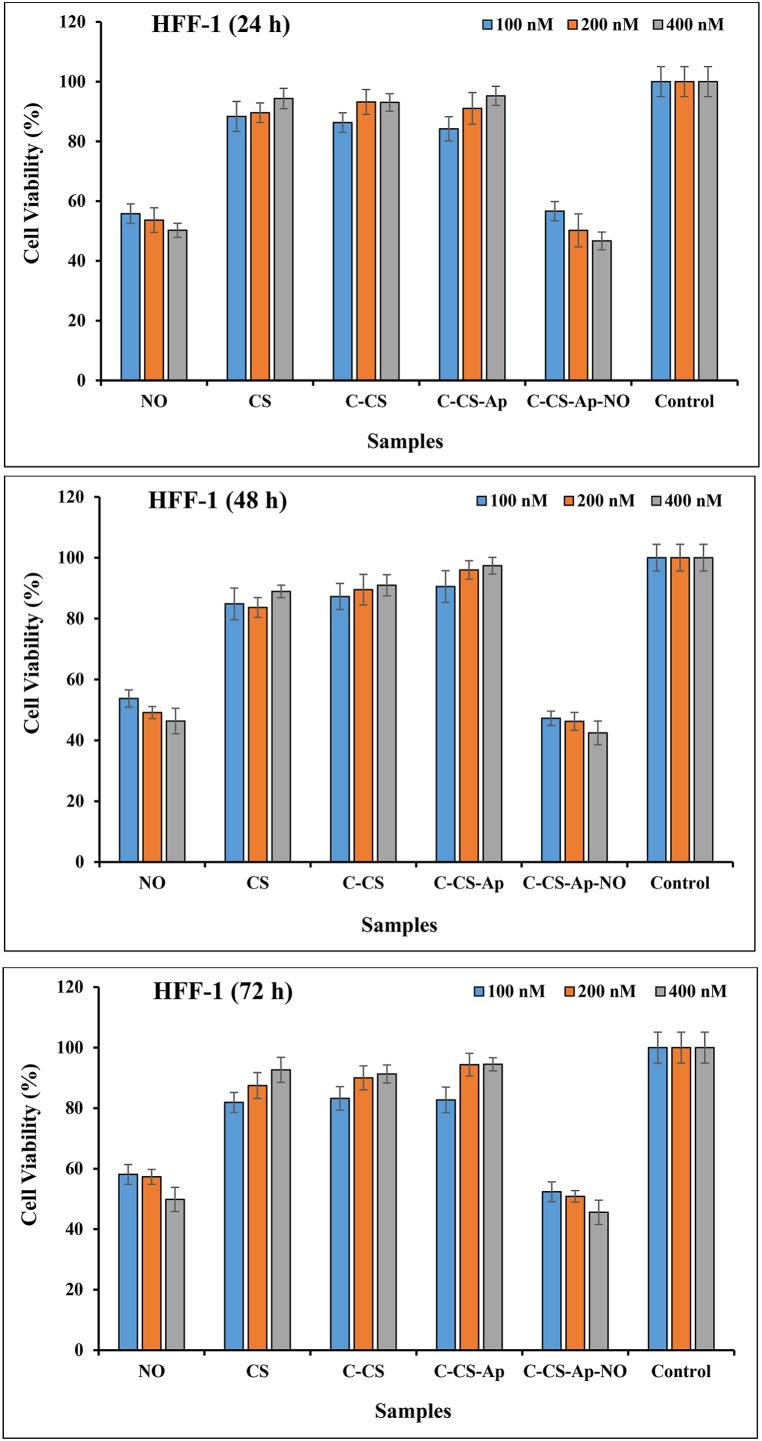
Effects of CS, C-CS, Ap-C-CS, Ap-C-CS-NO, and pure NO nano-complexes on cell viability of MCF-7 and HFF-1 cell lines after 24 h, 48 h, and 72 h of treatment with various concentrations (100 nM. 200 nM, 400 nM) of samples. The excellent cancer cell growth inhibition is exhibited for the Ap-C-CS-NO specimen. On the other hand, low cytotoxicity is attained for C-CS (for cancer cells) and Ap-C-CS (for normal cells) which is indicating excellent biocompatibility, biosafety, and biodegradability of the synthetic nano-complex.

The cytotoxic value and cancer cell growth inhibition performance of CS, C-CS, Ap-C-CS, Ap-C-CS-NO, and pure NO specimens with 100 nM, 200 nM, and 400 nM concentrations were investigated on MCF-7 (as cancer cell) and HFF-1 (as normal cell) cells applying the MTT assay. This test was performed after 24 h, 48 h, and 72 h of treatment with samples (Fig. 7). Generally speaking, the results are indicated the presence of a linear correlation between increasing the concentrations of Ap-C-CS-NO, Ap-C-CS, and pure NO samples and rising their toxic effect on the examined cancer cell line while the toxicity amounts were reversed for CS and C-CS formulations by increasing the concentration amounts. As indicated in Fig. 7, significant cytotoxicity was observed for the Ap-C-CS-NO formulation after 72 h at 400 nM (19.84 ± 1.87 % cell viability) concentration on MCF-7 cells which contained chemotherapeutic agents. Other two concentrations including 100 nM (40.5 ± 3.11 % cell viability) and 200 nM (30.65 ± 2.01 % cell viability) had considerable toxicity on cancer cell line after 24 h and 72 h of treatment whereas the cell viability values were 56.64 ± 3.21 % (100 nM) and 50.83 ± 1.89 % (200 nM) for HFF-1 cell line at the same times. These data are demonstrating that Ap-C-CS-NO nano-complex has a remarkable cytotoxicity effect on breast cancer cells than normal cells. According to Fig. 7, the 50 % maximal inhibitory concentration (IC50) of Ap-C-CS-NO is 100 nM for three post-treatment times in MCF-7 cell line whereas it is obtained at 200 nM for HFF-1 cell lines which is a few cytotoxicity for normal cells. The second cytotoxic sample on cancer cell line was pure NO; as 46.51 ± 2.19 %, 34.53 ± 1.93 %, and 48.51 ± 1.98 % of cell viability was observed after treatment with 400 nM concentration of pure NO at 24 h, 48 h, and 72 h of post-treatment respectively, while those values were 50.24 ± 2.35 % (24 h), 46.36 ± 4.2 % (48 h), and 49.82 ± 3.99 % (72 h) for normal cells at the same concentration. By comparing the cell viability results of Ap-C-CS-NO with pure NO, it is crystal clear that the Ap-C-CS-NO sample had better cancer cell growth inhibition potency than pure NO which could be owing to controlled drug release behavior and Ap-mediated internalization of nano-complex in cancer cells [15,19]. Therapeutic agent delivery using nanoparticles might decline drug waste and more importantly drug side effects. Additionally, the cell viability values of the Ap-C-CS sample were revealed that cell toxicity increase in cancer cells by raising its concentration which is due to EGFR-blocking ability of Ap which, in turn, leads to inducing cell death in cancer cells. As a matter of fact, since EGFR is overexpressed on the surface of cancer cells such as breast cancer cells, its blocking could lead to inhibition of cancer cell growth [18]. As can be seen in Fig. 7, the cell viability value was 73.03 ± 2.99 % after 24 h of treatment with 400 nM of Ap-C-CS while this value was 95.25 ± 3.2 % for normal cells at the same conditions. Hence, Ap-C-CS nano-complex can inhibit cancer cell growth and leads to cell death at the end. On the other hand, the cell viability values of CS and C-CS specimens have been indicated excellent biocompatibility, biosafety, and less cytotoxicity, especially by increasing their concentrations. As the cell viability values were 83.53 ± 1.98 % and 85.32 ± 3.27 % for cancer cell line after 24 h of treatment with 400 nM concentrations of CS and C-CS nano-complexes, respectively. Moreover, excellent biocompatibility, biosafety, and biodegradability were attained for normal cells in comparison to cancer cells; as the normal cells treated with CS and C-CS samples have been shown 94.35 ± 3.42 % and 93.05 ± 2.91 % of cell viability for 400 nM concentration after 24 h of post-treatment, respectively. These data have been presenting that Ap-conjugated C-CS could effectively bind to EGFR and consequently, leads to receptor-mediated internalizations of C-CS. Then, this C-CS can act as nutrition for cells since it is a kind of biopolymer with a high level of biocompatibility [27,44]. Our results are in line with a study which has conducted by Pina et al. in which they used an aptamer-modified CS for active targeting drug delivery to induce cancer cell death [45]. Additionally, our outputs are inconsistent with research study which has done by Zavareh et al., in which they applied an aptamer-conjugated CS-based nanocarrier for delivery of fluorouracil to cancer cells [46].

### 3.8 Cellular uptake

**Fig. 8:**
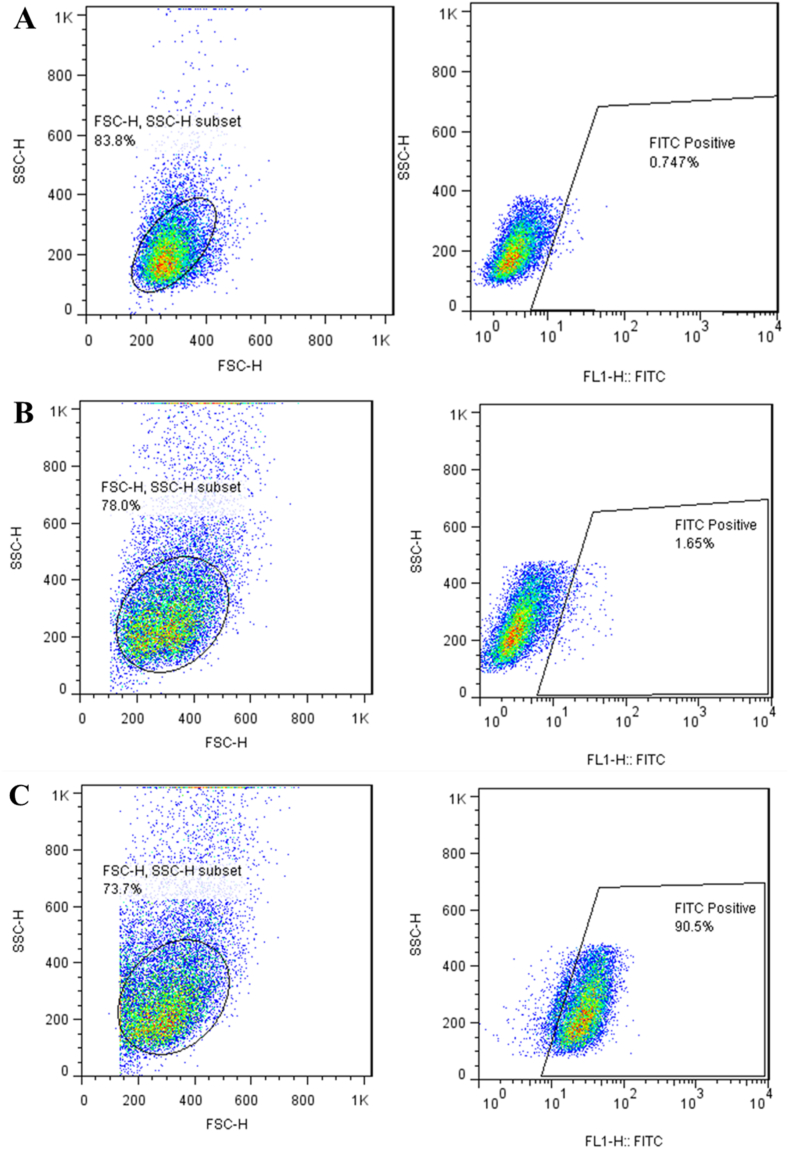
Cellular uptake of pure CS nanoparticle (B) and Ap-C-CS nano-complex (C) after 5 h of treatment on MCF7 cell line. A graph is control. The fluorescence intensity of MCF-7 cells showed after treatment with pure CS nanoparticles was acquired 1.65 % on cancer cells while it remarkably increased to more than 50 folds (90.5 %) on cancer cells after treatment with Ap-C-CS nanocarrier at the same circumstances.

The cellular internalization efficiency of FITC-labeled-pure CS and -Ap-C-CS nano-complex was investigated on MCF-7 cancer cells using flow cytometry. 100 nM concentration of samples was tested on cancer cells within 5 h. Fig. 8 is indicating the outcomes of Ap-decorate CS nanoparticles. The enhanced cellular uptake of Ap-C-CS in MCF7 breast cancer cell line verifies the potent efficiency of the Ap-equipped nanocarrier as an effective targeted DDS. The fluorescence intensity of MCF-7 cells showed after treatment with pure CS nanoparticles (B) was acquired 1.65 % on cancer cells while it remarkably increased to more than 50 folds (90.5 %) on cancer cells after treatment with Ap-C-CS nanocarrier at the same circumstances. Generally speaking, these data have been demonstrating that Ap-equipped CS nanoparticles can intensively enhance cellular internalization by even more than 50 times in breast cancer cells which is a considerable achievement in targeted cancer therapy [18]. Moreover, results show that this nano-complex can be a promising candidate for efficient DDS since Aps do not result in primary or acquired resistance in patients and potentially attach to EGFR on the surface of cancer cells [20,21].

## 4 Conclusion

In summary, a novel CS-based nano-complex modified with carboxyl and functionalized with DNA-Apt (as targeting and therapeutic agent) was designed and synthesized for the effective delivery of NO into EGFR-positive breast cancer cells. The synthetics were characterized via various analytical devices such as FT-IR, ^1^H NMR, DLS, Zeta potential, TGA, SEM, and SEM to evaluate the qualitative and quantitative aspects of CS derivatives. Nano-metric size (less than 100 nm), excellent drug loading capacity (about 25 %), and sustained drug release manner (less than 35 % release within 8 h) were measured for nano-complex which are appropriate for suing as effective DDS. Moreover, the cell viability assay was carried out to investigate cancer inhibition potency of synthetics; its results have shown the great ability of nano-complex in inhibition of breast cancer cell growth and inducing apoptosis in them. Ap and NO had also the synergistic effects on suppression of cancer cells and reducing their viability.

## CRediT authorship contribution statement

**Maryam Rahmani Kheyrollahi:** Writing – review & editing, Writing – original draft, Visualization, Validation, Funding acquisition, Formal analysis, Conceptualization. **Javad Mohammadnejad:** Writing – review & editing, Visualization, Validation, Supervision, Software, Resources, Methodology, Investigation, Funding acquisition, Formal analysis. **Akram Eidi:** Visualization, Validation, Supervision, Project administration, Methodology, Investigation. **Hanieh Jafary:** Visualization, Validation, Supervision, Software, Funding acquisition, Formal analysis.

## Declaration of competing interest

The authors declare that they have no known competing financial interests or personal relationships that could have appeared to influence the work reported in this paper.
